# Evaluation of DNA extraction kits and phylogenetic diversity of the porcine gastrointestinal tract based on Illumina sequencing of two hypervariable regions

**DOI:** 10.1002/mbo3.312

**Published:** 2015-11-05

**Authors:** Katharina Burbach, Jana Seifert, Dietmar H. Pieper, Amélia Camarinha‐Silva

**Affiliations:** ^1^ Institute of Animal ScienceUniversity of HohenheimStuttgartGermany; ^2^ Microbial Interactions and Processes Research GroupHelmholtz Centre for Infection ResearchBraunschweigGermany

**Keywords:** DNA extraction, feces, ileum digesta, Illumina amplicon sequencing, porcine, T‐RFLP

## Abstract

A robust DNA extraction method is important to identify the majority of microorganisms present in environmental microbial communities and to enable a consistent comparison between different studies. Here, 15 manual and four automated commercial DNA extraction kits were evaluated for their efficiency to extract DNA from porcine feces and ileal digesta samples. DNA yield, integrity, and purity varied among the different methods. Terminal restriction fragment length polymorphism (T‐RFLP) and Illumina amplicon sequencing were used to characterize the diversity and composition of the microbial communities. We also compared phylogenetic profiles of two regions of the 16S rRNA gene, one of the most used region (V1–2) and the V5–6 region. A high correlation between community structures obtained by analyzing both regions was observed at genus and family level for ileum digesta and feces. Based on our findings, we want to recommend the FastDNA ^™^ SPIN Kit for Soil (MP Biomedical) as a suitable kit for the analyses of porcine gastrointestinal tract samples.

## Introduction

The gastrointestinal tract is a complex ecosystem harboring a huge variety of microorganisms that influence the metabolism of nutrients and health of the host (Henderson et al. 2013). For instance, the degradation of complex dietary carbohydrates by gut microbes is an important source of energy and fermentation products that may stimulate immunity of the host. Factors like aging, host genetics, diet, environmental conditions, and medical treatments define the composition of the intestinal microbial community (Ley et al. 2008; Looft et al. 2014; Zhao et al. 2015). Previous studies of the porcine gut microbiota have focused on the influence of feeding strategies and antibiotic treatments on the community (Rist et al. 2013; Holman and Chenier 2014). However, recently, the pig has been recognized as a suitable animal model for human‐related research and is therefore of increasing interest (Heinritz et al. 2013).

The analysis of microbial community structures is typically performed through culture‐independent methods. Insights into phylogenetic composition of the porcine intestinal microbiota were obtained by using different approaches such as clone libraries (Leser et al. 2002), terminal restriction fragment length polymorphism (T‐RFLP) and quantitative PCR (qPCR) (Metzler‐Zebeli et al. 2010; Pedersen et al. 2013a), microarrays (Arnal et al. 2014), and high‐throughput sequencing technologies (Illumina and 454‐pyrosequencing) (Lamendella et al. 2011; Holman and Chenier 2014; Looft et al. 2014; Pajarillo et al. 2014). DNA extraction from intestinal samples has a crucial effect on the determination of community composition of the intestinal microbiota. Combined with the phylogenetic diversity, the diverse morphological and physicochemical properties of bacterial cells might affect the extraction. The efficient DNA extraction is challenging and requires some effort (Ariefdjohan et al. 2010; Maukonen et al. 2012).

A classical and inexpensive method of DNA extraction uses a phenol–chloroform mixture to purify DNA (Kirby 1956). This protocol is rather time‐consuming and demands hazardous chemicals that even inhibit downstream amplification reactions (Katcher and Schwartz 1994). Today, a wide range of commercial DNA extraction kits are available and are easy to use. Aside from differences in the protocols, the commercial kits have some general steps in common: cell lysis, washing, and DNA capture. Cell lysis occurs either through chemical lysis and/or by mechanical cell disruption with bead beating. In most cases, the released DNA is captured on a silica matrix in the presence of a solution with high concentration of salt. In automated systems, silica‐cladded magnetic beads are commonly used. Since PCR inhibitors like humic substances, polysaccharides, bile salts, and hemoglobin may be coextracted with DNA and affect downstream PCR application (Tsai and Olson 1992; Akane et al. 1994; Lantz et al. 1997; Monteiro et al. 1997), some DNA extraction kits include a PCR inhibitor removal step to avoid these complications.

Standardization of methods is an important step to gain reliable knowledge on bacterial community profiles. In the last years, some research consortia were formed as initiatives to investigate the earth microbiome (EMP) (Gilbert et al. 2011), human microbiome (HMP) (Human Microbiome Project Consortium, 2012), and human gut metagenome (MetaHit) (Qin et al. 2010). Such large‐scaled projects have to deal with multiple samples, whose analysis and interpretation need efficient techniques and standardized methods. A general protocol was suggested for studies of the HMP, using the MoBio PowerSoil^™^ kit for the DNA extraction from intestinal samples (McInnes and Cutting 2010). Other studies compared different protocols of DNA extraction from intestinal samples source and the effect on downstream applications (Ariefdjohan et al. 2010; Maukonen et al. 2012; Sergeant et al. 2012; Claassen et al. 2013; Guo and Zhang 2013; Desneux and Pourcher 2014; Ferrand et al. 2014; Rubin et al. 2014). So far, there are no standardized molecular genetic methods for extracting DNA of the porcine gastrointestinal tract. In this study, we compared 15 manual and four automated commercial DNA extraction kits to extract DNA from porcine feces and ileal digesta. The DNA extraction protocols were evaluated based on yield and purity of extracted DNA. Bacterial community profiles were analyzed with terminal restriction fragment length polymorphism (T‐RFLP) and bacterial community composition by Illumina amplicon sequencing spanning the V1–2 and V5–6 regions of the 16S rRNA gene.

## Materials and Methods

### Sample collection

The animal experiment was approved by the Animal Welfare commission of the University of Hohenheim in accordance to the German Animal Welfare legislation (Lorz and Metzger 1999).

One feces and one ileal digesta samples were obtained from a pig, fed with temperature pretreated soybeans to study ileal digestibility of this animal feed. The pig was surgically fitted with a simple T‐cannula at the distal ileum (Li et al. 1993). For collection of the ileal digesta a plastic bag was attached to the barrel of the cannula. Freshly voided feces were taken without being in contact with the floor. The samples were immediately put on ice and stored at −80°C.

### DNA extraction

Samples were thawed on ice, homogenized, and 250 mg each were used to extract DNA with commercial kits (Table 1) according to the respective manufacturer's instruction with slight modification as follows: as suggested in the protocol of the FastDNA^™^ SPIN Kit for Feces and FastDNA^™^ SPIN Kit for Soil (MP Biomedical, Solon, OH, USA) an extended centrifugation for 15 min at 14,000*g* was performed after homogenization in the FastPrep^®^‐24 instrument (MP Biomedical) at 6 m/sec for 40 sec. DES (DNase/Pyrogen‐Free Water) was warmed up at 55°C prior to DNA elution from the SPIN^™^ Filter. No Proteinase K was added to the NukExPure RNA/DNA (Gerbion, Kornwestheim, Germany) protocol. When using the Precellys Soil DNA Kit (PEQLAB, Erlangen, Germany) lysis was performed using the FastPrep^®^‐24 instrument at 4 m/sec for 20 sec, followed by centrifugation at 17,000*g* for 5 min. Lysis with the protocols of the PowerLyzer^™^ PowerSoil^®^, PowerFecal^™^, and PowerSoil^®^ DNA Isolation Kit (Mo Bio Laboratories, Carlsbad, CA, USA) was in all three cases performed with FastPrep^®^‐24 at 6.5 m/sec for 45 sec. In case of DNA extraction from fecal material using the PowerLyzer^™^ PowerSoil^®^ and PowerSoil^®^ DNA Isolation Kit an additional centrifugation at 10,000*g* for 2 min was performed to achieve a pellet. The NucleoSpin^®^ Soil (Macherey‐Nagel, Düren, Germany) protocol was evaluated in four variations of two alternative lysis buffers, SL 1 and SL 2, and in combination with or without the Enhancer SX. Lysis was always achieved using a FastPrep^®^‐24 at 5 m/sec for 30 sec. To obtain clear supernatant after the lysis of fecal material centrifugation was performed twice for 2 min at 11,000*g*. The InnuPREP Stool DNA Kit (Analytik Jena, Jena, Germany) was used according to the manufacturer's instructions, whereas the innuSPEED Stool DNA Kit and innuSPEED Soil DNA Kit (Analytik Jena) required an additional centrifugation for 2 min at 13,000*g* after lysis using the FastPrep^®^‐24 instrument at 6 m/sec for 40 sec. The QIAamp^®^ DNA Stool mini kit (Qiagen, Hilden, Germany) was used according to the manufacturer's instruction.

**Table 1:** Evaluated DNA extraction kits

Kit no.	Name	Company	Procedure	Lysis	DNA capture	Elution (*μ*L)
K1	FastDNA^™^ SPIN kit for feces	MP Biomedical	Manual	BB	SM	50
K2	FastDNA^™^ SPIN kit for soil	MP Biomedical	Manual	BB	SM	50
K3	NukExPure RNA/DNA	Gerbion	Manual	CL	SM	50
K4	Precellys soil DNA kit	PEQLAB	Manual	BB	SM	80
K5	PowerLyzer^™^ PowerSoil^®^ DNA isolation kit	Mo Bio	Manual	BB	SM	70
K6	PowerFecal^®^ DNA isolation kit	Mo Bio	Manual	BB	SM	70
K7	PowerSoil^®^ DNA isolation kit	Mo Bio	Manual	BB	SM	70
K8	Nucleo Spin^®^ soil SL1	Macherey Nagel	Manual	BB	SM	50
K9	Nucleo Spin^®^ soil SL1+Enhancer SX	Macherey Nagel	Manual	BB	SM	50
K10	Nucleo Spin^®^ soil SL2	Macherey Nagel	Manual	BB	SM	50
K11	Nucleo Spin^®^ soil SL2+Enhancer SX	Macherey Nagel	Manual	BB	SM	50
K12	InnuPREP stool DNA kit	Analytik Jena	Manual	CL	SM	80
K13	InnuSPEED Stool DNA kit	Analytik Jena	Manual	BB	SM	80
K14	InnuSPEED Soil DNA kit	Analytik Jena	Manual	BB	SM	50
K15	QIAamp^®^ DNA Stool mini KIT	Qiagen	Manual	CL	SM	100
K16	Promega Maxwell^®^ 16 A	Promega	Automatic	CL	MB	100
K17	Promega Maxwell^®^ 16 B	Promega	Automatic	CL	MB	100
K18	Promega Maxwell^®^ 16 C	Promega	Automatic	BB + CL	MB	100
K19	Promega Maxwell^®^ 16 D	Promega	Automatic	BB + CL	MB	100

BB, bead beating; CL, chemical lysis; MB, magnetic beads; SM, silica membrane.

In addition, DNA was extracted using four variations of the Maxwell^®^ 16 FFPE Plus LEV DNA Purification Kit (Promega, Mannheim, Germany) with the automatic DNA extraction by the Maxwell^®^ 16 Instrument (Promega). For the extraction named Promega Maxwell 16 A (K16), 80 mg feces or ileal digesta were added to 400 *μ*L lysis buffer and mixed thoroughly by vortexing. Twenty‐five microliters of these solutions were loaded into the machine and eluted with 100 *μ*L nuclease‐free water. Promega Maxwell^®^ 16 B (K17) followed the same procedure but with 80 mg of source material in 800 *μ*L lysis buffer. For Promega Maxwell^®^ 16 C (K18) and Promega Maxwell^®^ 16 D (K19), 80 mg source material were thoroughly resuspended in 400 *μ*L lysis buffer, followed by a bead beating step using FastPrep^™^ Lysing Matrix D and 50 *μ*g beads of 0.5 mm diameter, respectively, in the FastPrep^®^‐24 instrument at 6 m/sec for 40 sec. One hundred microliters of these solutions were used for DNA extraction followed by an elution in 100 *μ*L nuclease‐free water.

### DNA quality and quantity

The concentration of extracted DNA (absorbance at 260 nm) and its purity (absorbance ratio 260/230 and 260/280) were measured using NanoDrop (Thermo Fisher Scientific, Waltham, Massachusetts, EUA). Integrity of extracted DNA was analyzed by gel electrophoresis.

### T‐RFLP analysis

16S rRNA gene fragments were amplified from DNA extracts using the primer set 27F, labeled at the 5′ end with 6‐carboxyfluorescein (FAM), and 1492R (Lane 1991). Polymerase chain reaction (PCR) was performed in a 50 *μ*L mixture containing 1 *μ*L of a 1:10 dilution of DNA template, 10 *μ*mol/L of each primer, and 10 *μ*L of *Taq* 5× Master Mix (New England Biolabs, Ipswich, MA, USA). The PCR was run under the following conditions: initial denaturation at 95°C for 4 min, followed by 30 cycles of denaturation at 95°C for 30 sec, annealing at 52°C for 30 sec, extension at 68°C for 30 sec, and a final extension step at 68°C for 10 min. The size of amplified PCR products was verified by 1% agarose gel electrophoresis and four replicates of each PCR were pooled for purification with the QIAquick PCR Purification Kit (Qiagen). Seventy ng of purified PCR products were digested with 5 U MspI (New England BioLabs) for 1 h at 37°C. Triplicates of 2 *μ*L digested PCR product were mixed with 17 *μ*L HiDi Formamid (Applied Biosystems, Foster City, CA, USA), 0.5 *μ*L Map Marker 1500‐ROX (BioVentures, Murfreesboro, TN, USA) and 0.5 *μ*L Tracking Dye (BioVentures), followed by denaturation at 95°C for 5 min and subsequent cooling on ice for 5 min before further analysis on an ABI 3130xI Genetic Analyzer (Applied Biosystems). The peaks of separated terminal restriction fragments (TRFs) were exported with a threshold of 50 and analyzed with GeneMapper^®^ v.4.1 (Applied Biosystems, Foster City, CA, USA). For further analysis TRFs beyond the range of 45–1400 bp were excluded. The abundance of each fragment was standardized by the total fragment area within each sample. Two replicate profiles were used to generate a consensus profile using the T‐align program (Smith et al. 2005).

### Illumina amplicon sequencing

Library preparation with PCR amplification, purification, and equimolar pooling of samples for amplicon sequencing of the V1–2 region of the 16S rRNA gene was performed as previously described (Camarinha‐Silva et al. 2014). For Illumina amplicon sequencing of the V5–6 region, the 807F and 1050R primers were used (Bohorquez et al. 2012). The reaction mixture (20 *μ*L) contained PrimeSTAR buffer (Clontech Laboratories, Mountain View, CA, USA), each deoxynucleoside triphosphate at a concentration of 2.5 mol/L, each primer at a concentration of 0.2 *μ*mol/L, 5% dimethyl sulfoxide (DMSO), 1 *μ*L of template DNA, and PrimeSTAR HS DNA polymerase (2.5 U, Clontech Laboratories). An initial denaturation at 95°C for 3 min was followed by 20 cycles of denaturation at 98°C for 10 sec, annealing at 51°C for 10 sec, extension at 72°C for 45 sec, and a final extension for 2 min at 72°C. One microliter of the PCR product was used on a second PCR (15 cycles), following the same PCR conditions, where the forward primer contains a six nucleotide (nt) barcode (Hamady et al. 2008) and a 2 nt GT linker (Meyer and Kircher 2010). Both primers contained a sequence complementary to the Illumina‐specific adapters at the 5′ ends (Camarinha‐Silva et al. 2014) (Table S1). A third PCR (10 cycles) was performed in a 50 *μ*L reaction under the same conditions as described previously using primers that integrate the sequence of Illumina multiplexing sequencing and Illumina index primers (Camarinha‐Silva et al. 2014). Amplicons were purified with QIAquick PCR Purification Kit, quantified with QuantiFluor dsDNA System (Promega), and equimolar ratios (30 ng) of amplicons were pooled. Libraries were sequenced using 250 bp paired end sequencing chemistry on an Illumina MiSeq platform. A total of 4.3 million sequence reads were quality filtered and assembled using the RDP pipeline (Wang et al. 2007). Sequences were excluded if they have any primer or barcode mismatch and an *N* character. Sequences were assigned to a specific sample via the barcode, aligned, and checked for chimeras using uchime and clustered into operational taxonomic units (OTU) at ≥97% similarity (Wang et al. 2007). Low‐abundance OTUs, if present in <5 samples in relative abundances lower than 0.01%, were removed. Sample K3 ileal digesta was discarded from the analysis due to low number of reads (<350).

A total of 474 and 995 phylotypes (V1–2 region, ileal digesta and feces samples, respectively), and 1366 and 5281 phylotypes (V5–6 region, ileal digesta and feces samples, respectively) were taxonomically assigned using the naïve Bayesian RDP classifier (Wang et al. 2007). Sequences were submitted to the European Nucleotide Archive (ENA) under accession number PRJEB9411 (http://www.ebi.ac.uk/ena/data/view/PRJEB9411). All samples analyzed with V1–2 region comprised more than 11,000 sequence reads, where the mean number of reads per sample was 35,026 ± 2042. In regard to V5–6 region, all samples have more than 25,000 reads being the mean number of reads per sample 67,325 ± 3550.

### Statistical analysis

T‐RFLP and Illumina amplicon sequencing data sets were statistical analyzed using PRIMER (v.6.1.16, PRIMER‐E; Plymouth Marine Laboratory, Plymouth, UK) (Clarke and Warwick 2001). Abundance data, obtained from T‐RFLP and Illumina amplicon analysis, were standardized by total and sample resemblance matrixes were generated using Bray–Curtis similarity coefficient. Community structures were explored by nonmetric multidimensional scaling (MDS) and hierarchical clustering. Phylotype richness (Pielou's evenness) and diversity (Shannon diversity) were analyzed using univariate measures through PRIMER. Significant differences in diversity were evaluated by analysis of similarity (ANOSIM) (999 permutations) and considered significantly different if *P* < 0.05.

A Mantel‐type test (Relate routine in Primer) was used to measure how closely related were the results of the two evaluated regions of the 16S rRNA gene at the genus level (Clarke and Warwick 2001). Rho values were significant if <5% of 999 permutations were greater than the real rho. Multivariate dispersion analysis was used to calculate the intravariation among the same DNA extraction analyzed with each 16S rRNA region where a low dispersion index point to a higher similarity within sample. Species responsible for observed differences were identified by similarity percentages (SIMPER) (Clarke and Warwick 2001).

Illumina amplicon sequencing heat maps were generated with heatmap.2 provided by gplots package (Venables et al. 2012) implemented in R (version 3.1.2) and depict all the families present in relative abundance higher than 1% in at least one of the 16S rRNA regions analyzed.

## Results and Discussion

### DNA yield and purity

**Figure 1:**
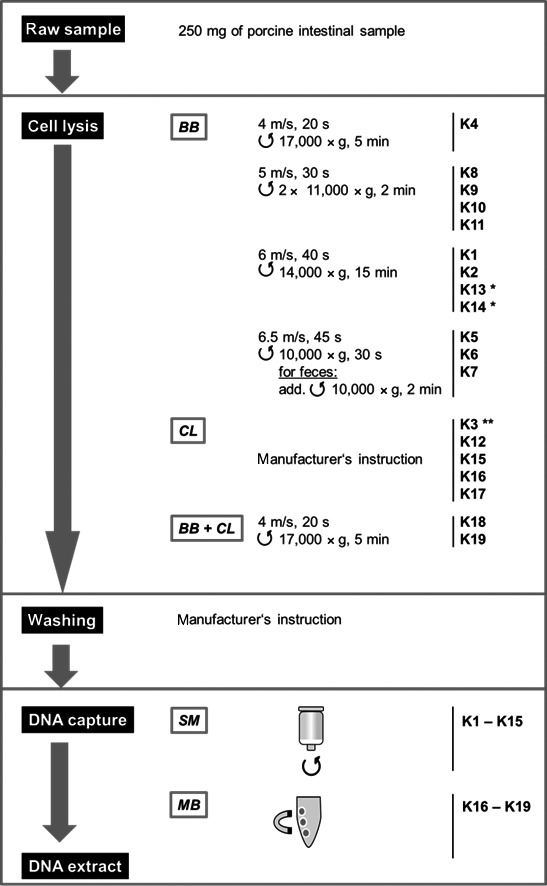
Flowchart comparing the main steps in DNA extraction protocols of the evaluated kits. BB, bead beating; BB + CL, chemical lysis with an additional bead beating treatment; CL, chemical lysis buffer; MB, magnetic beads; SM, silica membrane; 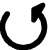 centrifugation, * for fecal sample an additional centrifugation step for 2 min with 13,000*g* was required to obtain a pellet, ** no Proteinase K was added.

Fifteen manual and four automated DNA extraction procedures were selected to extract total DNA from one pig fecal and one ileal digesta sample (Table 1, Fig. 1). We applied approximately 250 mg of sample for all commercial methods (K1–K15) and 80 mg for the automated procedure (K16–K19). Spectrophotometric measurements of the extracted DNA showed differences in quantity and quality according to the extraction kit and the sample source (Table 2). The DNA yield (ng DNA per mg source material) extracted from the fecal sample was higher than from the ileal digesta sample. For ileal digesta, DNA extraction kits K2, K3, K12, and K13 obtained the highest DNA yields with a mean yield of 16.6 ng/mg of sample. The other kits showed lower mean DNA yields of 1.5 ng/mg sample. For feces the highest yields were obtained with K1, K2, followed by K3 and K15. With these extraction kits there was a mean DNA yield of 47.9 ng/mg sample, where other kits yielded an average of 6.3 ng/mg sample. Previous studies have also analyzed DNA extraction using some of the same kits reported here in mice feces (Ferrand et al. 2014). The yields obtained for K1, K2, and K15 were similar to the ones reported in Table 2.

**Table 2:** Yield and purity of extracted DNA

	Ileal digesta				Feces			
	Yield^a^	Concentration (ng/*μ*L)	A_260/280_	A_260/230_	Yield^a^	Concentration (ng/*μ*L)	A_260/280_	A_260/230_
K1	2.1	12.1	6.1	0.0	54.8	301.0	1.9	0.6
K2	16.6	83.6	1.9	0.6	71.6	411.7	1.9	0.6
K3	20.5	108.8	2.2	1.2	34.7	178.2	2.2	1.2
K4	1.3	4.6	2.1	0.1	1.6	5.9	2.1	0.1
K5	0.5	2.1	0.7	−0.2	3.1	11.1	0.7	−0.2
K6	0.7	2.7	0.9	0.6	3.7	14.1	0.9	0.6
K7	0.2	0.8	0.5	−0.1	2.5	9.3	0.5	−0.1
K8	2.4	12.1	1.9	0.7	2.3	11.9	1.9	0.7
K9	1.6	8.1	2.2	0.6	6.4	34.6	2.2	0.6
K10	2.1	11.5	1.8	0.5	1.7	8.3	1.8	0.5
K11	2.4	13.5	1.8	0.3	11.7	60.6	1.8	0.3
K12	16.6	58.6	1.9	0.7	12.9	42.1	1.9	0.7
K13	12.8	42.8	2.1	0.9	10.2	31.0	2.1	0.9
K14	1.3	7.0	1.8	0.0	1.9	9.3	1.8	0.0
K15	3.2	8.1	2.0	1.5	30.5	71.0	2.0	1.5
K16	0.4	0.4	−4.5	0.1	7.3	5.8	−4.5	0.1
K17	0.9	0.7	4.1	0.0	7.0	5.6	4.1	0.0
K18	2.5	2.0	1.8	0.1	8.1	6.5	1.8	0.1
K19	1.3	1.0	1.9	0.1	14.8	11.8	1.9	0.1

a Yield: DNA (ng) per source material (mg).

Another important parameter that can affect the PCR‐based community profiling is the quality of extracted DNA. Some of the analyzed kits showed values lower than 1 for the A260/280 ratio (Table 2) that can be associated with protein contamination or some reagent used during the extraction (Hansen et al. 2007; Neary et al. 2014). These kits were also the ones giving low DNA yield from the ileal digesta samples. In this study, we used the A260/230 values to have a measure of non‐nucleic acid contaminations in each DNA extraction (Table 2). The results indicated non‐nucleic acid contaminations in each DNA extract (A260/230: ileal digesta = 0.4 ± 0.1; feces = 0.44 ± 0.1). These low values may be due to impurities, buffer salts, and/or residual guanidine that are commonly used in column‐based kits (Hansen et al. 2007; Neary et al. 2014). However, this was not affecting further DNA usage, as a 1:10 dilution was suitable for successful amplification of the 16S rRNA gene for all DNA extracts using either T‐RFLP or Illumina amplicon sequencing.

Overall, our results indicate that some kits show better yields, purity, and quality of the DNA extracted being more suitable for downstream use. These results were consistent with a study comparing DNA extraction kits for denaturing gradient gel electrophoresis (DGGE) that also concluded that DNA extraction efficiency is crucial for comprehensive reflection of microbial communities (Ariefdjohan et al. 2010).

### Comparing bacterial communities based on diversity

**Figure 2:**
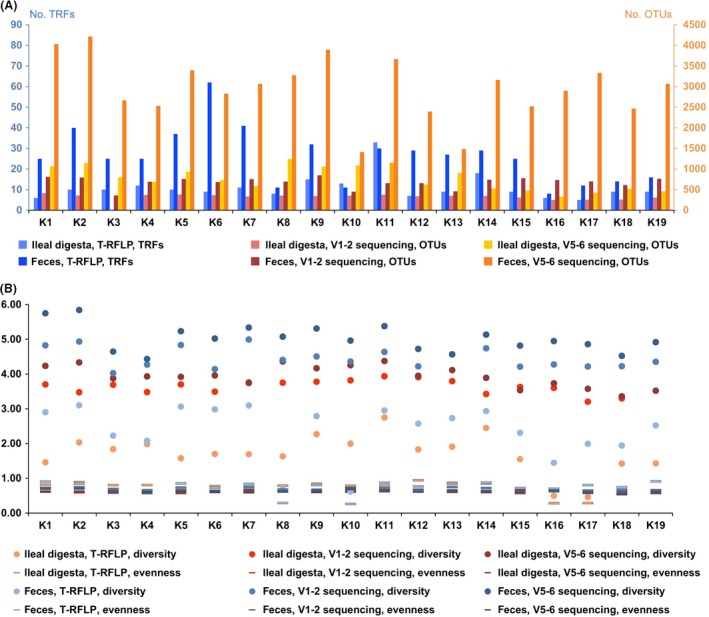
Numerical comparison of terminal restriction fragment length polymorphism (T‐RFLP) analysis and Illumina 16S rRNA gene amplicon sequencing. (A) Number of terminal restriction fragments (TRFs) and operational taxonomic units (OTUs) obtained. TRFs are plotted in blue on the left *y*‐axis and OTUs are plotted in red‐orange on the right. (B) Diversity indices of bacterial communities recovered by evaluated DNA extraction kits. Circle symbols show Shannon diversity and bars show Pielou's evenness. Diversity indices of fecal samples are plotted in shades of blue and ileal digesta samples in shades of red.

To compare bacterial communities recovered by the tested DNA extraction kits, T‐RFLP analysis and Illumina 16S rRNA amplicon sequencing were performed. There was a heterogeneous distribution in the mean numbers of TRFs and OTUs among the tested DNA extraction kits and type of sample (Fig. 2A).

This indicates a large variation in the capability of recovering a certain given bacterial community. The mean numbers of TRF and OTU units showed great standard deviation for DNA extractions from ileal digesta and feces samples. The mean TRF number of DNA extracted was 11 ± 1 and 26 ± 3 from ileal digesta and feces, respectively. Illumina amplicon sequencing of the V1–2 region of the 16S rRNA gene generates an average of 339 ± 11 OTUs (V5–6 region: 777 ± 66 OTUs) from ileal digesta and 682 ± 30 OTUs from feces (V5–6 region: 2964 ± 173 OTUs). The ileum digesta sample, extracted with the automated procedures showed the lowest number of OTUs, regardless of the 16S region analyzed, however, this was not observed for the feces sample where these procedures gave similar numbers of OTUs as the majority of the tested kits. In general sequencing of the 16S rRNA gene region V5–6 resulted in higher numbers of OTUs than sequencing the V1–2 region.

Regardless the kit used, higher numbers of TRFs and OTUs were obtained from fecal samples than from ileal digesta (Fig. 2A). This finding of reduced richness in ileal digesta is consistent with the study of Looft et al. who compared bacterial communities in different sections of porcine intestine (Looft et al. 2014).

The mean Shannon diversity across all DNA extraction kits and analyzing methods for ileal digesta was 3.1 ± 0.70. The top three DNA extraction kits based on the highest Shannon index, for T‐RFLP, are K9, K11, and K14. Although K11, K12, and K13 gave amplicons that indicated a higher diversity in case of the V1–2 region, amplicons obtained by K2, K8, and K11 extractions showed the highest diversity when analyzing the V5–6 region (Fig. 2B). The lowest diversity was observed in the DNA extractions using the automated kit. For feces the mean Shannon diversity across all DNA extraction kits and both T‐RFLP and Illumina amplicon sequencing was 3.9 ± 0.81. The highest diversity was observed with K2, K5, and K7 with T‐RFLP and V1–2 amplicon sequencing analysis, while V5–6 region showed more diversity when K1, K2, and K11 extractions had been used (Fig. 2B). The differences observed on the Shannon diversity index result suggest that the fecal sample analyzed with the different kits do not demonstrate similar diversity profile.

**Figure 3:**
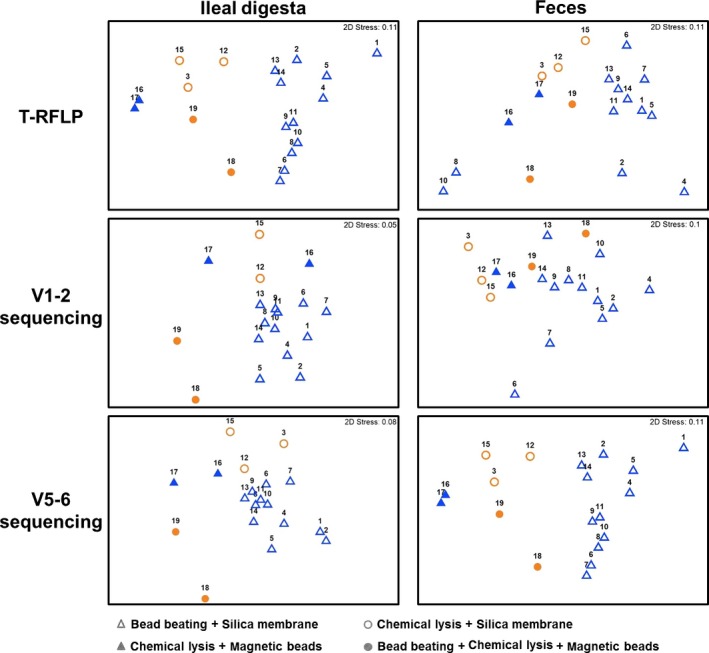
Nonmetric multidimensional scaling (MDS) plot comparing the global bacterial community of all samples analyzed with terminal restriction fragment length polymorphism (T‐RFLP) and Illumina amplicon sequencing. Abundance data were standardized with Bray–Curtis similarity algorithm. Symbols refer to cell lysis and DNA capture technique of the DNA extraction protocol.

The differences in global composition of bacterial communities of ileal digesta and feces samples evaluated after DNA extraction using different kits are depicted in Figures 3 and S1. A main group, that show similar microbial communities, contains 12 DNA extraction kits that combine cell lysis with bead beating and DNA capture with a silica membrane spin filter. This group clusters together with some outliers in T‐RFLP analysis and V1–2 amplicon sequencing of feces material. The clustering shown in Figure 3 suggests an effect of lysis and DNA recovering on the recovered microbial community composition. Significant differences in bacterial communities with respect to DNA extraction kits using different lysis approaches were observed in the ileum digesta extractions (T‐RFLP: *R *= 0.67, *P *= 0.001; V1–2: *R *= 0.8, *P* = 0.001; V5–6: *R *= 0.66, *P* = 0.002). There were no significant differences in the communities recovered from feces extractions, where the *R*‐statistics were close to 0 and *P* > 0.1, however at 60% similarity the dendrograms of feces samples extracted with different methods showed 8 (T‐RFLP), 3 (V1–2), and 2 (V5–6) clusters of samples (Fig. S1). Kits that have a bead beating step were clustering together suggesting that different species abundance patterns were found consistently in those groups (Fig. S1). Independently of the analysis method the bacterial community of the ileal digesta of K9 and K11 clustered together (Figs. 3 and S1) showing that the addition of enhancer SX in the NucleoSpin^®^ Soil (Macherey‐Nagel) protocol leads to the extraction of similar bacterial communities even if different lysis buffers are used. Also, this addition resulted in higher DNA yields and Shannon diversity. These results suggest that it may be worth to add the enhancer when this kit is chosen for DNA extraction from porcine intestine samples. Such a variation of the DNA extraction protocol was previously recommended by Desneux and Pourcher (2014).

### Comparing bacterial communities based on phylogenetic structure

A recent review article lists several studies surveying porcine intestine samples using different 16S rRNA gene regions (Kim and Isaacson 2015). One of the most used region to study bacterial communities of porcine gut and feces samples is the V1–3 region (Kim and Isaacson 2015). The majority of these studies used 454‐pyrosequencing to analyze porcine gut contents and feces samples at different life stages of the animal. Although the V5 region has low resolution, one study analyzed colon and cecum samples using Illumina sequencing spanning this region (Pedersen et al. 2013b). Other studies characterize these niches using different regions (V4–5, V4–6, and V5–6) (Cousin et al. 2012; Riboulet‐Bisson et al. 2012; Upadrasta et al. 2013). Overall different variable regions are used across the world by different research groups; their choice mainly depends on their experience and knowledge. A comparison of the different variable regions has shown that none of them gives equal results (Claesson et al. 2010). Each 16S rRNA gene region gives different phylogenetic accuracy and the direct comparison of studies is hindered by the absence of using a consensus region (Hamady and Knight 2009).

In the present study, DNA extraction protocols were compared in regard to the bacterial community profiles obtained with Illumina amplicon sequencing spanning the V1–2 and V5–6 regions of the 16S rRNA gene. A new Illumina amplicon approach based on a previous study (Camarinha‐Silva et al. 2014) was developed using the V5–6 region. A high correlation between community structures obtained by analyzing both regions was observed at genus (*Rho* = 0.875, *P* = 0.001) and family level (*Rho *= 0.862, *P* = 0.001) for ileal digesta and also for feces samples (genus: *Rho* = 0.935, *P* = 0.001; family: *Rho *= 0.921, *P* = 0.001) suggesting that both regions are appropriate to analyze the porcine gastrointestinal tract.

Ileal digesta samples showed microbial communities where most of the members belonged to the phyla *Firmicutes*,* Proteobacteria*,* Bacteroidetes*, and *Fusobacteria*. Due to its similarity to the major phyla occurring in the human gut (*Firmicutes* and *Bacteroidetes*) (Karlsson et al. 2013), the pig has already been used as an animal model for humans (Heinritz et al. 2013). Bacterial families belonging to these phyla and present in abundances higher than 1%, in at least one of the 16S rRNA community analyses are depicted in Figure 3. Regardless of the different extraction methods tested, ileal sample analyzed within V1–2 and V5–6 regions showed 67–68% similarity at genus and 71–72% at family levels. Some variances were observed in the community composition detected with each variable region and within DNA extraction procedures. Only one family was identified in the ileal digesta sample using the V5–6 region that was not detected with V1–2 region. *Rhodospirillaceae*, a family of the *α‐Proteobacteria*, was not detected in the porcine intestine of other studies, but it is known to be a member of communities in soybean‐planted soils (Ge et al. 2014). Since pigs were fed with soybeans the recovered *Rhodospirillaceae* might be of feed origin.

The relative abundances of the most abundant families are shown in Figure 3. Despite the fact that bead beating improves the detection of Gram‐positive bacteria, this effect was not uniformly observed at genus level of ileal digesta sample, across the extractions methods that use mechanical lysis, leading to the differences in the detection of the families that account for 63–73% of total community abundance. Similarity percentage analyses indicated that OTUs belonging to uncultured *Veillonellaceae*,* Streptococcus*,* Clostridium* cluster XI, and *Actinobacillus* were responsible for the observed differences between the extractions. The genus *Clostridium XI* of the *Peptostreptococcaceae* family was not equally identified by all extraction kits, with K2 giving the highest abundance (24–26%) and K18 the lowest (4–5%) (Fig. S2). *Streptococcus* was detected in higher abundances using the K5 extraction method (29–34%) but almost not presented in the samples extracted with K15 (<0.7%) (Fig. S2). Both genera were previously found in the lumen of the ileum in abundances close to 5% (Looft et al. 2014). As previously recognized by Sergeant and colleagues it is still uncertain which protocol will give us the most correct description of community structure (Sergeant et al. 2012). At genus level, ileal digesta extracted with K2 showed the highest average community similarity (73%) and the lowest dispersion index (0.1), indicating little difference between the microbial communities analyzed by both regions.

**Figure 4:**
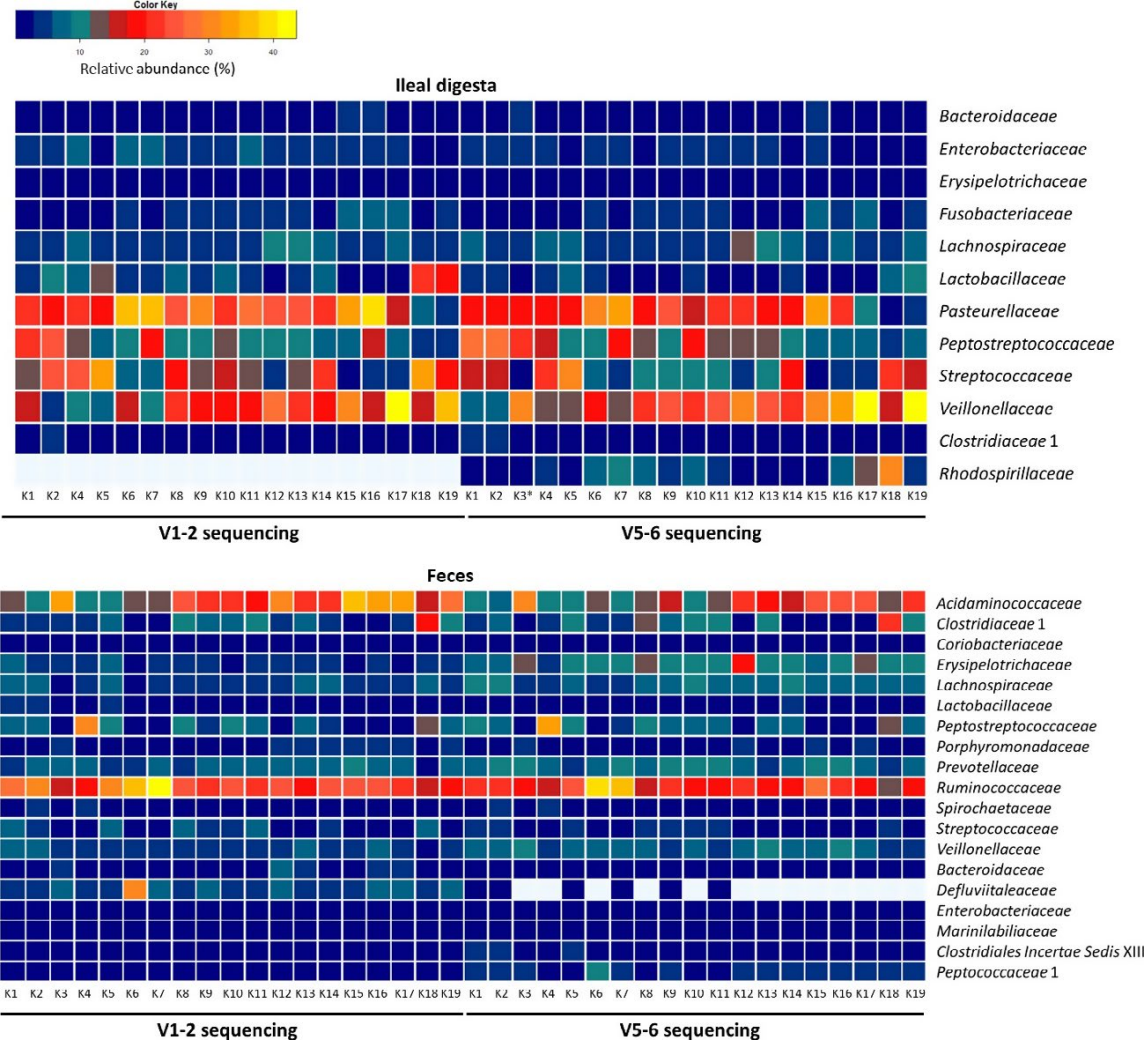
Heat map plots depicting the bacterial communities of the ileal digesta and fecal sample at family level. Operational taxonomic units detected by Illumina sequencing in a relative abundance higher than 1%, in at least one of the 16S rRNA regions, are assigned to bacteria families and listed on the right of the heat maps. The relative abundance of these bacteria families in each DNA extract is coded according to the color key on the top of the figure. K3* indicates missing data for sequencing 16S rRNA gene region V1–2 of DNA extracted with K3 from ileal digesta, data were excluded because of low read numbers.

DNA extraction from feces revealed 69–71% and 75–77% similarity in community structure within each 16S region at genus and family level. All evaluated DNA extracts from porcine feces showed the presence of five bacterial phyla (*Firmicutes*,* Bacteroidetes*,* Proteobacteria*,* Spirochaetes*, and *Actinobacteria*) that occurred in a relative abundance higher than 1%, for at least one of the 16S rRNA regions analyzed (Fig. 4). This result is in agreement with previous microbiome studies of porcine feces (Lamendella et al. 2011; Holman and Chenier 2014; Looft et al. 2014). A higher representation of *Peptostreptococcaceae* (K4) was observed in feces, once compared to the other extractions kits. K4, K5, and K2 have shown the highest similarity percentage (67%, 65%, and 62%, respectively) in the bacterial community structure analyzed with both regions, showing the closeness of the samples within the same extraction and analyzed with different 16S rRNA regions. *Clostridiales Incertae Sedis* XIII and *Peptococcaceae* 1 were only observed in average abundances lower than 0.09% using the V1–2 region. Microorganisms belonging to the families *Bacteroidaceae*,* Defluviitaleaceae*,* Enterobacteriaceae*, and *Marinilabiliaceae* were only detected in average abundances lower than 0.9% with V5–6 region.

Regardless the region, bacterial community profiles, of ileal digesta sample, obtained with DNA extraction protocols using bead beating and compared with the ones of chemical lysis extraction revealed that the group of OTUs contributing for 24–26 average dissimilarity belongs to *Firmicutes*. This is consistent with previous comparative studies of DNA extraction protocols that showed that bead beating improves the detection of *Firmicutes* (Wu et al. 2010; Henderson et al. 2013; Santiago et al. 2014). *Firmicutes* members, like *Blautia*, were detected in higher relative abundance when using bead beating to disrupt cells compared to chemical lysis. Thus, both type of lysis detect differently some groups of microorganisms.

A previous study on the effect of different DNA extraction conditions on the community structure showed that mechanical cell lysis by bead beating increases relative abundance of Gram‐positive bacteria even if this effect did not occur to be uniform within a genus (Sergeant et al. 2012). Such underestimation was found by all 16S rRNA gene sequencing analysis at the phyla level with K5 (PowerLyzer^™^ PowerSoil^®^DNA Isolation Kit). Underestimation of one bacterial cell wall type might be crucial for obesity studies that often use the comparison of *Firmicutes* and *Bacteroidetes* ratio (Pedersen et al. 2013a). In a previous work, evaluating DNA extractions from porcine intestinal samples, the usage of a modified QIAamp^®^ DNA Stool Mini Kit (Qiagen) was suggested (Li et al. 2003), despite all conclusions lead to similar DGGE results and bacterial detection (12 clones) irrespective of the lysis approach. Also, this kit was compared to a bead beating protocol where severe bead conditions were used (bead beating was performed twice for 2 min) and these conditions are normally not applied in commercial kits.

To study bacterial communities of complex intestinal environments the DNA extraction protocol of choice should be able to extract DNA from different types of source material with a similar efficiency. One evaluated DNA extraction kit that revealed unequal efficiency of ileal digesta and feces sample source was K7, PowerSoil^®^ DNA Isolation Kit (Mo Bio Laboratories), which is used in two large scale microbiome studies, EMP and HMP (McInnes and Cutting 2010). Surprisingly, in the present study, the lowest DNA yield from ileal digesta was obtained using this kit, resulting in low numbers of TRFs and OTUs. However, the diversity indices of the K7 fecal DNA extract showed a good efficiency compared to the results obtained from other kits.

Thus, according to our findings optimizations of DNA extraction are recommendable to ensure comprehensive representation of bacterial communities. Caution should be taken when data are compared across different studies as differences in community structure were observed depending on the extraction method chosen. Finally, based on the results obtained with both samples types and among all tested DNA extraction kits, the FastDNA^™^ SPIN Kit for Soil (K2) from MP Biomedical emerged as the most suitable one for analyzing microbial diversity of the porcine gastrointestinal tract. It gives a high species richness, good DNA yield and quality, and it shows, at genus level, high similarity percentage of bacterial community within both regions for the porcine ileal digesta and feces samples and a low dispersion index.

## Conclusion

This study compared 19 different DNA extraction protocols to identify the most suitable one for a comprehensive investigation of the porcine gastrointestinal microbiota. The criteria chosen were based on the DNA yield and purity as well as on the diversity indices and phylogenetic compositions identified by T‐RFLP and Illumina sequencing. A high correlation between the community structures obtained V1–2 and V5–6 16S rRNA regions at family and genus levels for ileal digesta and feces samples showed that both are suitable for future studies. The evaluation of the results showed that the FastDNA^™^ SPIN Kit for Soil (K2) was the most efficient for bacterial community characterization and should be the kit of choice in future DNA‐based analyses of porcine gastrointestinal samples.

## Conflict of Interest

None declared.

## Supporting information


**Table S1.** Primers used in this study for amplicon sequencing of the V5–6 region.
**Figure S1.** Dendrogram for hierarchical clustering of analyzed samples based on Bray–Curtis similarity.
**Figure S2.** Relative abundance of genus of interest across the ileum digesta sample analyzed with different extraction kits.
