# Supplementary material for: Evaluation of DNA extraction kits and phylogenetic diversity of the porcine gastrointestinal tract based on Illumina sequencing of two hypervariable regions
Source: Microbiologyopen. 2015 Nov 5;5(1):70–82. doi: 10.1002/mbo3.312 (PMC4767427; doi:10.1002/mbo3.312)
Supplement: Supplementary file 1 — Table S1. Primers used in this study for amplicon sequencing of the V5–6 region. Figure S1. Dendrogram for hierarchical clustering of analyzed samples based on Bray–Curtis similarity. Figure S2. Relative abundance of genus of interest across the ileum digesta sample analyzed with different extraction kits. [file MBO3-5-070-s001.docx]

**Supplementary material**

**Evaluation of DNA extraction kits and phylogenetic diversity of the porcine gastrointestinal tract based on Illumina sequencing of two hypervariable regions**

Katharina Burbach^1^, Jana Seifert^1^,Dietmar H. Pieper^2^, Amélia Camarinha-Silva^1#^

^1^ Institute of Animal Science, University of Hohenheim, Stuttgart, Germany

^2^Microbial Interactions and Processes Research Group, Helmholtz Centre for Infection Research - HZI, Inhoffenstrasse 7, 38124 Braunschweig, Germany

Table S1. Primers used in this study for amplicon sequencing of the V5-6 region.

| Primer name | Primer Sequence (5'-3') | Reference |
| --- | --- | --- |
| IlluBC1 | ACACTCTTTCCCTACACGACGCTCTTCCGATCTTTGATTGTGGATTAGATACCCBRGTAGTC | This study |
| IlluBC2 | ACACTCTTTCCCTACACGACGCTCTTCCGATCTTTAATGGTGGATTAGATACCCBRGTAGTC |  |
| IlluBC3 | ACACTCTTTCCCTACACGACGCTCTTCCGATCTAACCAGGTGGATTAGATACCCBRGTAGTC |  |
| IlluBC4 | ACACTCTTTCCCTACACGACGCTCTTCCGATCTAAGACCGTGGATTAGATACCCBRGTAGTC |  |
| IlluBC5 | ACACTCTTTCCCTACACGACGCTCTTCCGATCTAATGGAGTGGATTAGATACCCBRGTAGTC |  |
| IlluBC6 | ACACTCTTTCCCTACACGACGCTCTTCCGATCTACGTTGGTGGATTAGATACCCBRGTAGTC |  |
| IlluBC7 | ACACTCTTTCCCTACACGACGCTCTTCCGATCTAGTCTCGTGGATTAGATACCCBRGTAGTC |  |
| IlluBC8 | ACACTCTTTCCCTACACGACGCTCTTCCGATCTCAACTTGTGGATTAGATACCCBRGTAGTC |  |
| IlluBC9 | ACACTCTTTCCCTACACGACGCTCTTCCGATCTCAGTAAGTGGATTAGATACCCBRGTAGTC |  |
| IlluBC10 | ACACTCTTTCCCTACACGACGCTCTTCCGATCTCCTAACGTGGATTAGATACCCBRGTAGTC |  |
| IlluBC11 | ACACTCTTTCCCTACACGACGCTCTTCCGATCTCGAGAGGTGGATTAGATACCCBRGTAGTC |  |
| IlluBC12 | ACACTCTTTCCCTACACGACGCTCTTCCGATCTCGCATAGTGGATTAGATACCCBRGTAGTC |  |
| IlluBC13 | ACACTCTTTCCCTACACGACGCTCTTCCGATCTGCTCCAGTGGATTAGATACCCBRGTAGTC |  |
| IlluBC14 | ACACTCTTTCCCTACACGACGCTCTTCCGATCTGGTTATGTGGATTAGATACCCBRGTAGTC |  |
| IlluBC15 | ACACTCTTTCCCTACACGACGCTCTTCCGATCTAACGCAGTGGATTAGATACCCBRGTAGTC |  |
| IlluBC16 | ACACTCTTTCCCTACACGACGCTCTTCCGATCTAACTGCGTGGATTAGATACCCBRGTAGTC |  |
| IlluBC17 | ACACTCTTTCCCTACACGACGCTCTTCCGATCTAGTTGGGTGGATTAGATACCCBRGTAGTC |  |
| IlluBC18 | ACACTCTTTCCCTACACGACGCTCTTCCGATCTATAACTGTGGATTAGATACCCBRGTAGTC |  |
| IlluBC19 | ACACTCTTTCCCTACACGACGCTCTTCCGATCTATAGACGTGGATTAGATACCCBRGTAGTC |  |
| IlluBC20 | ACACTCTTTCCCTACACGACGCTCTTCCGATCTATATTGGTGGATTAGATACCCBRGTAGTC |  |
| IlluBC21 | ACACTCTTTCCCTACACGACGCTCTTCCGATCTCAAGAGGTGGATTAGATACCCBRGTAGTC |  |
| IlluBC22 | ACACTCTTTCCCTACACGACGCTCTTCCGATCTCAATTCGTGGATTAGATACCCBRGTAGTC |  |
| IlluBC23 | ACACTCTTTCCCTACACGACGCTCTTCCGATCTCAGGCCGTGGATTAGATACCCBRGTAGTC |  |
| IlluBC24 | ACACTCTTTCCCTACACGACGCTCTTCCGATCTCCAACGGTGGATTAGATACCCBRGTAGTC |  |
| IlluBC25 | ACACTCTTTCCCTACACGACGCTCTTCCGATCTGTTCCGGTGGATTAGATACCCBRGTAGTC |  |
| Illu Rev | GTGACTGGAGTTCAGACGTGTGCTCTTCCGATCTAGYTGDCGACRRCCRTGCA |  |


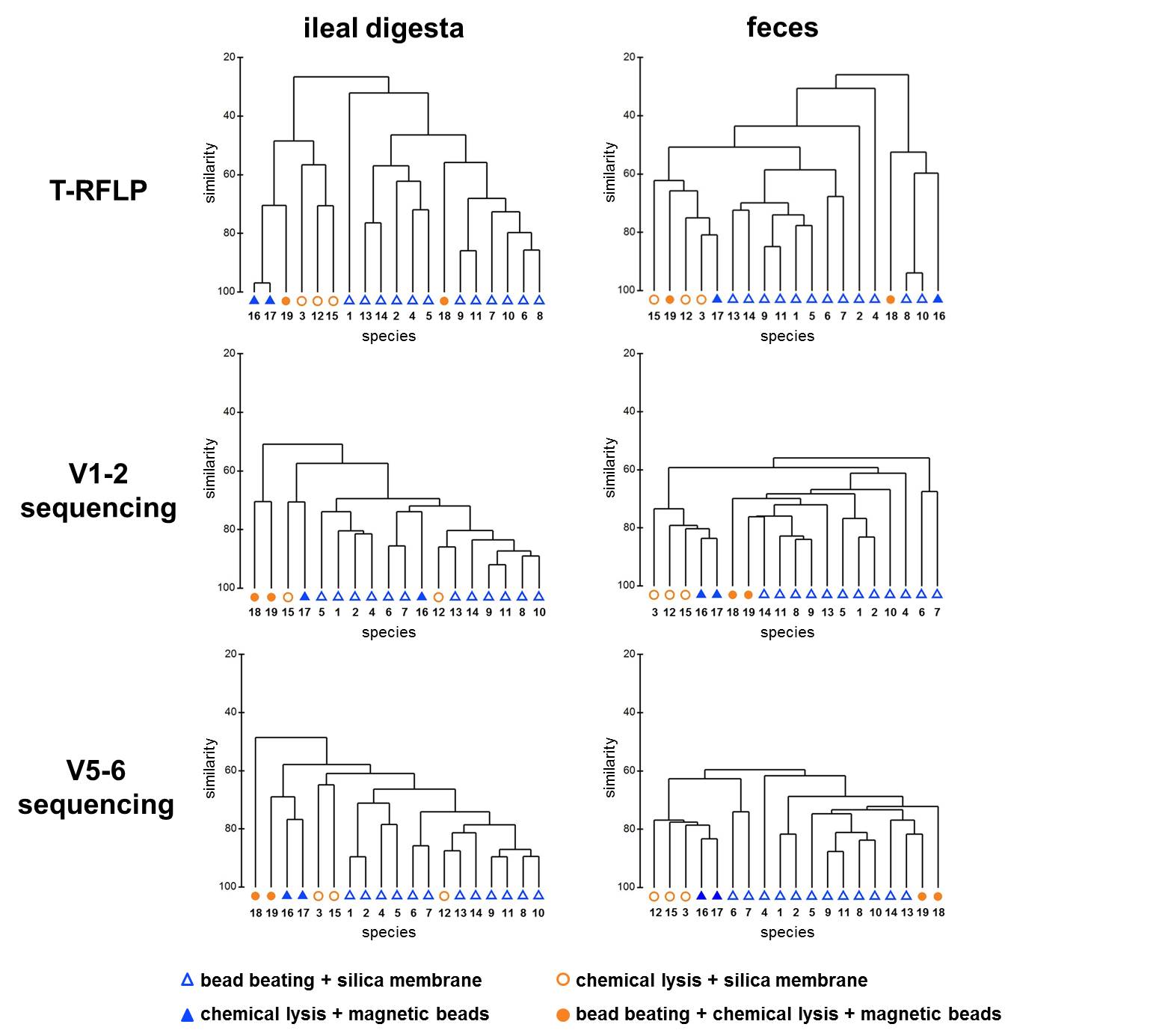


Figure S1. Dendrogram for hierarchical clustering of analysed samples based on Bray –Curtis similarity.


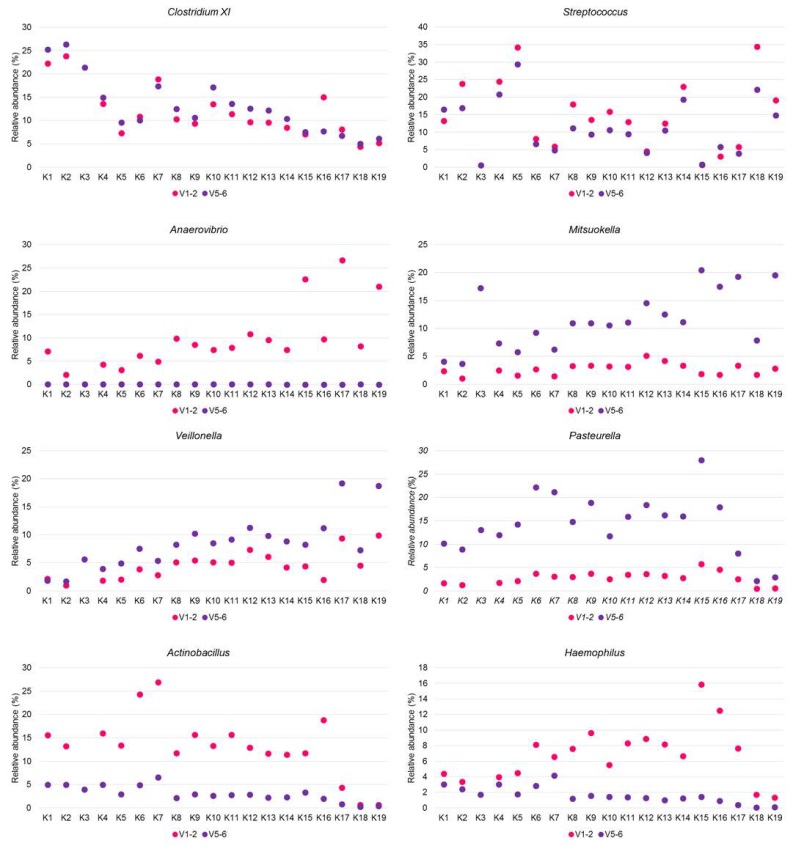


Figure S2. Relative abundance of genus of interest across the ileum digesta sample analysed with different extraction kits.
